# S-TIR-ring up TLR7 and TLR9: signaling domain substitutions clarify the TLR paradox

**DOI:** 10.1172/JCI198981

**Published:** 2025-11-03

**Authors:** Roser Tachó-Piñot, Carola G. Vinuesa

**Affiliations:** The Francis Crick Institute, London, United Kingdom.

## Abstract

In systemic lupus erythematosus (SLE), autoimmunity often develops toward self nucleic acids. The nucleic acid receptors TLR7 and TLR9, which sense RNA and DNA, respectively, are critical for the generation of pathogenic autoimmune antibodies. Despite similarities in their downstream signaling cascades, these receptors play opposing roles in most mouse lupus models: TLR7 promotes disease, while TLR9 provides protection — an observation often referred to as “the TLR paradox.” To understand the basis of this dichotomy, Leibler et al. created genetically edited lupus-prone mice in which TLR7 receptors express the TLR intracellular signaling domain (TIR) that corresponds to TLR9, or vice versa. Their results revealed that the TIR domains contribute to the receptors’ opposing roles in SLE, shedding light into the TLR paradox in autoimmunity.

Systemic Lupus Erythematosus (SLE) is an autoimmune disease characterized by loss of immune tolerance to self antigens, often targeting nucleic acids. Antibodies bound to nuclear antigens contribute to SLE pathogenesis by facilitating TLR7-mediated activation of immune cells, such as plasmacytoid dendritic cells (pDCs) and neutrophils, and via immune complex deposition (1). These mechanisms drive inflammation and tissue injury, with renal involvement being a major complication; lupus nephritis represents one of the most severe manifestations of SLE (1).

## TLR7 and TLR9 in SLE

TLR7 and TLR9 are innate sensors for single-stranded (ss) RNA and CpG-rich DNA, respectively. These transmembrane pattern-recognition receptors encounter their ligands in the endosomes of activated immune cells, such as pDCs and B cells (2). Upon ligand binding, TLR7 and TLR9 recruit the adaptor MyD88, which initiates a signaling cascade leading to IRF5 activation, which is critical to trigger autoimmunity (2–6).

While these innate sensing pathways play a role in the response to infection, mouse models of SLE have shown that TLR7 and TLR9 are also central to the production of autoantibodies to RNA and DNA-containing targets, respectively (7, 8). Indeed, TLR7 overexpression induces systemic autoimmunity in mice, and gain-of-function mutations in *TLR7* are a cause of human lupus (9–14). Yet, despite apparent convergence in the signaling pathways downstream of TLR7 and TLR9, TLR9 plays a paradoxical role in SLE.

## The TLR9 paradox

In mouse models of SLE, B cell–intrinsic TLR9 expression is necessary for the generation of pathogenic autoantibodies against DNA (8), and in BALB/c mice specifically, TLR9 expression is required for lupus-like disease (15). However, lupus-prone MRL/lpr mice lacking TLR9 develop more severe disease than their TLR9-sufficient counterparts, with heightened immune activation, worsened renal pathology, and increased mortality (7). Together, these findings support an overall protective role for TLR9 in the MRL/lpr mouse model of SLE.

To understand the mechanisms underlying TLR9’s protective role, previous work from the Shlomchik lab demonstrated that TLR9’s protective function in SLE occurs via a scaffold-mediated mechanism (16). This scaffold mechanism could be fulfilled by TLR9^K51E^, a point mutant that cannot bind ligand and therefore fails to trigger TLR9 signaling (16). Put simply, these experiments showed that TLR9 confers protection just by being there. TLR9’s scaffold mechanism may involve competition with TLR7 to bind the chaperone Unc93b1, which is necessary to transport TLR7 and TLR9 to their endosomal compartments (17, 18). The corollary is that increased TLR7 trafficking in the absence of competition with TLR9 may worsen autoimmunity. However, this scaffold function of TLR9 cannot fully explain its protective action, as it does not account for why signaling downstream of TLR7 can drive disease, whereas the same pathway downstream of TLR9 cannot (7, 8, 16). Rather, protective MyD88-independent signaling downstream of TLR9 may carry out this component of protection, as indicated by experiments with a MyD88-disrupting TLR9^P915H^ variant (16). Together, these previous findings suggested that qualitative differences in the signaling cascades downstream of TLR7 and TLR9 may underlie their opposing roles in SLE.

## Divergent TIR domain–mediated signaling between TLR7 and TLR9

**Figure 1:**
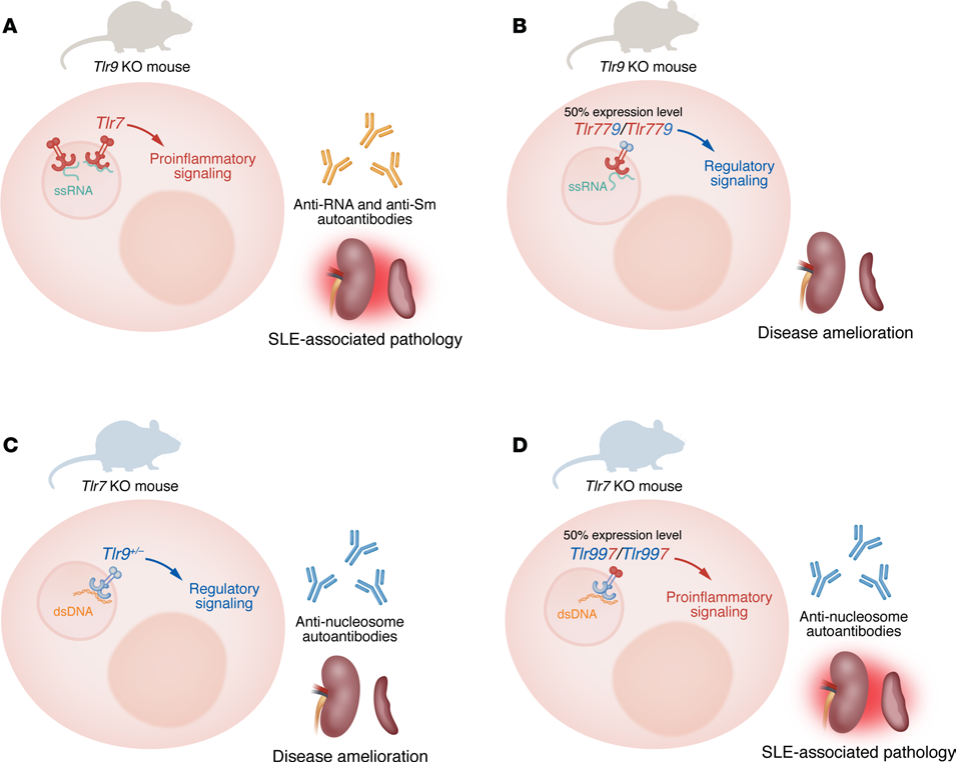
Differences in qualitative signaling downstream of TLR7 and TLR9 TIR domains. (**A**) In the lupus-prone MRL/lpr background, *Tlr7* WT *Tlr9*^–/–^ mice show SLE-associated pathology, with splenomegaly and nephritis, and high titres of anti-RNA and anti-Smith (anti-Sm) autoantibodies. (**B**) To evaluate the differential role of the TLR7 and TLR9 TIR domains, the *Tlr9* TIR domain was introduced into the *Tlr7* locus (termed *Tlr779*). *Tlr779* mice expressed reduced levels of TLR779 protein relative to expression level of TLR7 in **A**. Due to *Tlr7* being X-linked, heterozygous mice were not deemed appropriate dosage controls. However, these experiments showed that TLR779 domain led to reduced spleen and lymph node cellularity, nephritis, and anti-RNA and anti-Sm autoantibodies when compared with *Tlr7* WT mice in the MRL/lpr *Tlr9*^–/–^ background. Moreover, the TLR9 TIR domain induced the expression of genes involved in negative regulation, pointing to potential mechanisms underlying this effect. In the converse experiments, relative to *Tlr9* WT mice on the MRL/lpr *Tlr7*^–/–^ background (**C**), introduction of the *Tlr7* TIR domain into the *Tlr9* locus (**D**) led to increased expression of proinflammatory signalling molecules, which translated into aggravation of disease. Unexpectedly, antinucleosome antibodies were similar between *Tlr997* and *Tlr9*^+/–^ mice. Note that, while TLR997 showed reduced expression relative to TLR9 WT expression levels, this was controlled by comparing to heterozygous *Tlr9*^+/–^ mice.

In this issue of *JCI*, Leibler et al. extended the Shlomchik lab’s prior findings, establishing that differences in the TIR domains of TLR7 and TLR9 explain their opposing contributions to autoimmunity in a mouse model of lupus (19). To dissect this mechanism, the group genetically engineered mice to incorporate the *Tlr9* TIR domain into the *Tlr7* endogenous locus (*Tlr779* mice), or the *Tlr7* TIR domain into the *Tlr9* endogenous locus (*Tlr997* mice) (Figure 1).

*Tlr779* mice — in which the *Tlr7* TIR domain was replaced with the *Tlr9* TIR domain — showed disease amelioration when compared with WT *Tlr7* counterparts in the lupus-prone MRL/lpr *Tlr9^–/–^* background. TLR779 largely localized to the same endosomes as WT TLR7; however, TLR779 expression in B cells was 50% of that of WT TLR7 (19). Since *Tlr7* is an X-linked gene, a substantial fraction of immune B cells and pDCs express a single functional *Tlr7* allele (20). As such, *Tlr7* heterozygous mice were not deemed appropriate dosage controls. These experiments lacked TLR7 dosage controls, despite TLR7 gene dosage being a well established driver of autoimmunity (21). Nevertheless, in the MRL/lpr *Tlr9*^–/–^ background, *Tlr779* and *Tlr7^–/–^* mice were similarly protected from disease, suggesting that signaling through the TLR9 TIR domain does not produce the pathogenic effects that signaling through the TLR7 TIR domain does (19).

In these experiments, *Tlr779* did not function as a null allele, as it was able to signal and induce an immune phenotype distinct from both WT and *Tlr7*-KO mice in the MRL/lpr *Tlr9^–/–^* background. This immune phenotype included an increase in the percentage of CD19^+^ B cells in the spleen, with intermediate levels of age-associated B cells, germinal center B cells, and naive and effector CD4^+^ T cells and an increase in the frequency of CD4^+^ memory B cells in *Tlr779* mice when compared with WT *Tlr7* and *Tlr7^–/–^* mice (19).

To understand the potential differences in TIR domain signaling, Leibler et al. compared the response to the TLR7 ligand CL097 in *Tlr779* and *Tlr7* WT B cells, in an MRL/lpr *Tlr9^–/–^* background. B cells expressing TLR779 induced higher expression of some regulatory genes, including *Ets1*, when compared to B cells expressing WT TLR7 (19). This finding aligns with previous reports from the group proposing that TLR9’s TIR domain could induce protective signaling (16).

Leibler et al. (19) next undertook a reciprocal approach to confirm these conclusions, generating *Tlr997* mice, whereby the *Tlr9* TIR domain was replaced with the *Tlr7* TIR domain, in an MRL/lpr *Tlr7^–/–^* background (Figure 1). TLR997 maintained its endosomal localization but was expressed at lower levels than its WT TLR9 counterpart. To correct for the lower TLR997 expression, *Tlr9^+/–^* mice were included as TLR9 dose controls.

In B cells from MRL/lpr *Tlr7^–/–^* mice, *Tlr997* and *Tlr9^+/–^* induced broadly similar NF-kB nuclear translocation upon CpG stimulation. However, *Tlr997* B cells showed enrichment in the expression of genes related to type 1 IFN signaling when compared with *Tlr9^+/–^* B cells. Conversely, stimulated *Tlr9^+/–^* B cells showed higher expression of genes related to IL-4 and IL-13 signaling, and to negative regulation of NF-kB, MAPK, and IFN signaling pathways. Together, these findings support the group’s hypothesis that differential signaling downstream of TLR7 and TLR9 is encoded by their TIR domains.

To understand the functional effect of these differences, Leibler et al. (19) stimulated B cells from MRL/lpr *Tlr7^–/–^* mice bearing *Tlr997* or *Tlr9^+/–^*. In *Tlr997* mice, CpG stimulation induced higher plasmablast differentiation, suggesting that the signaling downstream of TLR7 TIR domain is a stronger inducer of this process. This is in line with an earlier report by another research group showing that TLR7-BCR costimulation promotes higher B cell survival and differentiation into plasmablasts compared with TLR9-BCR engagement (22).

Differences in B cell responses evoked by the different TIR domains were paralleled by changes in SLE severity. In an MRL/lpr *Tlr7^–/–^* background, *Tlr997* mice showed increased spleen weight and nephritis scores relative to *Tlr9^+/–^* mice. *Tlr997* mice also showed SLE-associated immune changes, such as increased age-associated B cells and decreased marginal zone B cells. As expected, and consistent with lack of RNA binding via the extracellular TLR9 domain, *Tlr997* mice had reduced anti-Smith antibodies (which are highly specific to SLE) and anti-RNA autoantibodies. Unexpectedly, *Tlr997* mice did not show increased anti-nucleosome antibodies compared with *Tlr9^+/–^* mice, although the presence of a TLR7 TIR domain linked to the DNA-binding TLR9 extracellular domain should promote such a response.

## Future work and open questions

The work by Liebler et al. (19) supports the hypothesis that qualitative differences in signaling downstream of the TIR domains of TLR7 and TLR9 underscore their opposing roles in autoimmunity. This work also raises unresolved questions. For instance, the absence of an increase in anti-nucleosome autoantibodies in *Tlr997* mice remains unexplained. This further suggests that anti-DNA/RNA antibodies do not contribute to the readouts evaluated by the authors — namely splenomegaly and histological evidence of nephritis.

While the experiments performed here explore the role of the TLR7 and TLR9 TIR domains independently by knocking out the reciprocal gene in their experiments (*Tlr9^–/–^* or *Tlr7^–/–^* background), the potential functional interactions between these receptors in the same cell and their signaling cascades remain unexplored. Along these lines, while transcriptional differences downstream of TLR7 and TLR9 TIR domains were investigated here, the underlying mechanisms were not. A likely contributor is different interacting protein partners of each TIR domain based on amino acid sequence differences. Whether such interactions are conserved between mice and humans also remains to be explored, since sequence conservation of TLR7 and TLR9 TIR domains is 93% and 87% homologous, respectively, between species, with four serine and one threonine residues present in human TLR9 TIR domain but not in its mouse ortholog.

Additional factors that could influence the opposing functions of TLR7 and TLR9 in SLE include differences in the concentration and nature of their ligands or receptor expression patterns, although these were controlled in the experimental settings employed by Leibler et al. (2, 19). Together, the experiments performed by Leibler et al. (19) show that the differences in the TIR domains of TLR9 and TLR7 contribute to their contrasting roles in systemic autoimmunity.

## Funding support

This work was supported by the Francis Crick Institute (CC2228), which receives its core funding from Cancer Research UK, the UK Medical Research Council and the Wellcome Trust; and has also received funding by a Royal Society Wolfson Fellowship, a Wellcome Trust Discovery grant and a Lupus Research Alliance Global team award to CGV.

## Version 1 11/03/2025

Electronic publication
